# Distribution of influenza virus types by age using case-based global surveillance data from twenty-nine countries, 1999-2014

**DOI:** 10.1186/s12879-018-3181-y

**Published:** 2018-06-08

**Authors:** Saverio Caini, Peter Spreeuwenberg, Gabriela F. Kusznierz, Juan Manuel Rudi, Rhonda Owen, Kate Pennington, Sonam Wangchuk, Sonam Gyeltshen, Walquiria Aparecida Ferreira de Almeida, Cláudio Maierovitch Pessanha Henriques, Richard Njouom, Marie-Astrid Vernet, Rodrigo A. Fasce, Winston Andrade, Hongjie Yu, Luzhao Feng, Juan Yang, Zhibin Peng, Jenny Lara, Alfredo Bruno, Doménica de Mora, Celina de Lozano, Maria Zambon, Richard Pebody, Leticia Castillo, Alexey W. Clara, Maria Luisa Matute, Herman Kosasih, Nurhayati, Simona Puzelli, Caterina Rizzo, Herve A. Kadjo, Coulibaly Daouda, Lyazzat Kiyanbekova, Akerke Ospanova, Joshua A. Mott, Gideon O. Emukule, Jean-Michel Heraud, Norosoa Harline Razanajatovo, Amal Barakat, Fatima el Falaki, Sue Q. Huang, Liza Lopez, Angel Balmaseda, Brechla Moreno, Ana Paula Rodrigues, Raquel Guiomar, Li Wei Ang, Vernon Jian Ming Lee, Marietjie Venter, Cheryl Cohen, Selim Badur, Meral A. Ciblak, Alla Mironenko, Olha Holubka, Joseph Bresee, Lynnette Brammer, Phuong Vu Mai Hoang, Mai Thi Quynh Le, Douglas Fleming, Clotilde El-Guerche Séblain, François Schellevis, John Paget, Global Influenza B Study group, Binay Thapa, Sangay Zangmo, Guy Vernet, Patricia Bustos, Patricio Loyola, Joanna Ellis, Antonino Bella, Maria Rita Castrucci, Gulzhan Muratbayeva, Julia Guillebaud, Laurence Randrianasolo, Ausenda Machado, Pedro Pechirra, Jeffery Cutter, Raymond Tzer Pin Lin

**Affiliations:** 1 Netherlands Institute for Health Services Research (NIVEL), Otterstraat 118-124, 3513 CR Utrecht, The Netherlands; 2 Instituto Nacional de Enfermedades Respiratorias “Dr. Emilio Coni”, Santa Fe, Argentina; 3 Vaccine Preventable Diseases Surveillance Section, Health Policy Protection branch, Office for Health Protection, Department of Health, Woden, Canberra, Australia; 4 Public Health Laboratory, Department of Public Health, Ministry of Health, Thimphu, Bhutan; 5 Ministry of Health, Brasilia, DF Brazil; 6 Virology Department, Centre Pasteur of Cameroon, Yaoundé, Cameroon; 7 Sección Virus Respiratorios, Instituto de Salud Pública de Chile, Santiago, Chile; 8 Division of Infectious Disease, Key Laboratory of Surveillance and Early-warning on Infectious Disease, Chinese Center for Disease Control and Prevention, Beijing, China; 9 National Influenza Center, Ministry of Health, San José, Costa Rica; 10 Instituto Nacional de Investigacion en Salud Publica (INSPI), Centro de Referencia Nacional de Influenza y otros Virus Respiratorios, Guayaquil, Ecuador; 11 National Influenza Center, Ministry of Health, San Salvador, El Salvador; 12 Respiratory Virus Unit, Public Health England, London, Colindale UK; 13 Respiratory Diseases Department, Public Health England, London, Colindale UK; 14 National Influenza Center, Ministry of Health, Guatemala City, Guatemala; 15 US Centers for Disease Control, Central American Region, Guatemala City, Guatemala; 16 National Influenza Center, Ministry of Health, Tegucigalpa, Honduras; 17 US Naval Medical Research Unit No.2, Jakarta, Indonesia; 18 National Influenza Center, National Institute of Health, Rome, Italy; 19 National Center for Epidemiology, Surveillance and Health Promotion, National Institute of Health, Rome, Italy; 20 Department of Epidemic Virus, Institut Pasteur, Abidjan, Côte d’Ivoire; 21 Service of Epidemiological Diseases Surveillance, National Institute of Public Hygiene, Abidjan, Côte d’Ivoire; 22 National Center of Expertise, Committee of Consumer Right Protection, Astana, Kazakhstan; 23 Zonal Virology Laboratory, National Center of Expertise, Committee of Consumer Right Protection, Astana, Kazakhstan; 24 Centers for Disease Control and Prevention - Kenya Country Office, Nairobi, Kenya; 25 US Public Health Service, Rockville, Maryland USA; 26 National Influenza Center, Virology Unit, Institut Pasteur of Madagascar, Antananarivo, Madagascar; 27 National Influenza Center, Institut National d’Hygiène, Ministry of Health, Rabat, Morocco; 28 Institute of Environmental Science and Research, Wellington, New Zealand; 29 National Influenza Center, Ministry of Health, Managua, Nicaragua; 30 National Influenza Center, IC Gorgas, Panama City, Panama; 31 Department of epidemiology, National Institute of Health Doutor Ricardo Jorge, Lisbon, Portugal; 32 National Influenza Reference Laboratory, National Institute of Health Doutor Ricardo Jorge, Lisbon, Portugal; 33 Epidemiology and Disease Control Division, Ministry of Health, Singapore, Singapore; 34 Communicable Diseases Division, Ministry of Health, Singapore, Singapore; 35 Global Disease Detection, US-CDC, Pretoria, South Africa; 36 Zoonoses Research Center, Department of Medical Virology, University of Pretoria, Pretoria, South Africa; 37 Centre for Respiratory Diseases and Meningitis (CRDM), National Institute for Communicable Diseases, Johannesburg, South Africa; 38 School of Public Health, Faculty of Health Science, University of the Witwatersrand, Johannesburg, South Africa; 39 Istanbul University, Istanbul, Turkey; 40 L.V.Gromashevsky Institute of Epidemiology and Infectious Diseases National Academy of Medical Science of Ukraine, Reiv, Ukraine; 41 Epidemiology and Prevention Branch, Influenza Division, Centers for Disease Control and Prevention, Atlanta, GA USA; 42 National Institute of Hygiene and Epidemiology, Hanoi, Vietnam; 43 Independent scientist, Birmingham, UK; 44 Sanofi Pasteur, Lyon, France; 45 Department of General Practice & Elderly Care Medicine, Amsterdam Public Health Research Institute, VU University Medical Center, Amsterdam, the Netherlands

**Keywords:** Influenza, Age distribution, Influenza A virus, H3N2 subtype, Influenza A virus, H1N1 subtype, Influenza B virus, Meta-analysis

## Abstract

**Background:**

Influenza disease burden varies by age and this has important public health implications. We compared the proportional distribution of different influenza virus types within age strata using surveillance data from twenty-nine countries during 1999-2014 (N=358,796 influenza cases).

**Methods:**

For each virus, we calculated a Relative Illness Ratio (defined as the ratio of the percentage of cases in an age group to the percentage of the country population in the same age group) for young children (0-4 years), older children (5-17 years), young adults (18-39 years), older adults (40-64 years), and the elderly (65+ years). We used random-effects meta-analysis models to obtain summary relative illness ratios (sRIRs), and conducted meta-regression and sub-group analyses to explore causes of between-estimates heterogeneity.

**Results:**

The influenza virus with highest sRIR was A(H1N1) for young children, B for older children, A(H1N1)pdm2009 for adults, and (A(H3N2) for the elderly. As expected, considering the diverse nature of the national surveillance datasets included in our analysis, between-estimates heterogeneity was high (I^2^>90%) for most sRIRs. The variations of countries’ geographic, demographic and economic characteristics and the proportion of outpatients among reported influenza cases explained only part of the heterogeneity, suggesting that multiple factors were at play.

**Conclusions:**

These results highlight the importance of presenting burden of disease estimates by age group and virus (sub)type.

**Electronic supplementary material:**

The online version of this article (10.1186/s12879-018-3181-y) contains supplementary material, which is available to authorized users.

## Background

Several studies have shown that the disease burden and costs of influenza vary considerably by age group [1, 2]. Among adults, influenza is usually a self-limiting disease and the social costs are mainly due to loss of productivity and workdays and to absenteeism due to the need to care for family members [3]. At the extremes of age there is a much higher risk of complications, hospitalization and influenza-associated death [4, 5], especially among older people with underlying conditions such as chronic heart disease or diabetes [6]. In these age groups, the costs for society are mainly related to healthcare.

The influenza burden of disease varies substantially across seasons [1]. This depends on the circulating virus strains and, where vaccination campaigns are implemented, on the vaccine uptake rate as well. Overall, the disease burden of influenza is thought to be higher in seasons dominated by A(H3N2) or A(H1N1)pdm2009 influenza viruses, and lower in seasons where pre-pandemic A(H1N1) or influenza B account for the majority of cases [7, 8].

The dependency of the disease burden in a given season on circulating influenza virus (sub)types may be due to two factors that are complementary to each other. On one hand, influenza viruses may differ between one another in terms of their ability to induce a severe or complicated clinical illness, for instance by favouring bacterial co-infections, worsening of underlying conditions, or other life-threatening events. This hypothesis has been questioned by studies showing that there were only negligible differences in the age-adjusted clinical presentation and severity of the illness caused by different influenza virus types and subtypes [9–12]. On the other hand, the influenza viruses may differ between one another in how frequently they affect certain age groups. For instance, school-age children are more affected during influenza seasons dominated by pre-pandemic A(H1N1) and B virus strains than during A(H3N2)-dominated season [13].

The age distribution of influenza cases is likely to be affected by several other factors beyond the mix of circulating virus (sub)types , which may vary considerably in space and time. These include the country’s demographic and geographic characteristics, the implementation of vaccination campaigns against influenza, and past epidemics that affect the immunity of the different population age groups. In addition, how the influenza surveillance system is structured, the general perception of the severity of the season (for example, during the 2009 pandemic), and health care seeking behaviour may determine how many and what influenza cases are seen in each season and country. The availability of a global database with age-specific data enables an analysis of patterns across different settings and facilitates stronger conclusions, provided there is careful examination of how the age distribution of influenza cases fluctuated across countries and seasons, and investigating the influence of potential confounding factors.

Here, we compared the proportional distribution of different influenza virus (sub)types within age strata using the database of the Global Influenza B Study (GIBS).

## Methods

### Sources of data and definitions

The GIBS was launched in 2012 with the aim of increasing the scientific evidence needed to optimize the influenza prevention policies worldwide [14]. Experts from over fifty countries from all continents were invited to provide data on the weekly number of influenza cases reported to their national influenza surveillance system during recent years (ideally, from 2000 onwards) (N=948,646). Importantly, and in contrast to other global surveillance databases (e.g. FluNet) [15], the GIBS database also includes the age (exact age or age group) of each reported influenza case. From large countries we asked to provide data stratified at a sub-national level, if available (for brevity, we will use the term “country” to refer to a whole country or to a sub-national region of it hereinafter). Experts from participating countries were also requested to describe the main characteristics of their influenza surveillance system.

Country-specific geographic, demographic and socioeconomic information necessary for the analyses was derived from the US Central Intelligence Agency World Factbook website [16]. This includes information on the population age structure, the ageing index (defined as the number of people aged 65 years or older per hundred people aged 14 years or younger), the latitude of the population centroid (when available) or of the largest city, and the per capita gross domestic product (GDP). In terms of latitude, a country was considered as being situated in the Northern or Southern hemisphere or in the inter-tropical belt based on the latitude of its centroid/largest city.

### Statistical analysis

For brevity, we will describe the outcome variable by referring to “influenza”, and the method was applied to A(H1N1), A(H1N1)pdm2009, A(H3N2), and influenza B viruses separately; influenza cases that could not be classified in an unambiguous way to any of the above (for instance, those classified as “unsubtyped influenza A”) were not included in the analysis. The statistic used to describe the age distribution of influenza cases in each season and country is the Relative Illness Ratio (RIR) [17], which is defined as the ratio of the percentage of influenza cases in a given age group to the percentage of the general population belonging to the same age group. We considered the following age groups: 0-4 years (referred to as “young children” hereinafter), 5-17 years (“older children”), 18-39 years (“young adults”), 40-64 years (“older adults”), and 65+ years (“elderly”). A RIR was calculated for each of these five age groups in each influenza season in which there were at least 100 reported influenza cases overall; 95% confidence intervals (95% CI) were calculated by using an exact method based on the Poisson distribution [18].

When calculating a RIR, the null hypothesis is that the chance of being infected with influenza is the same for all people irrespective of their age, implying an age distribution of influenza cases perfectly equal to that of the general population and the RIR equal to 1 in each age group. Therefore, a RIR equal to 3 for influenza B in the age group 5-17 years would mean that, in that season, the proportion of influenza B cases aged 5-17 years was three times the proportion of the general population of the same age group, i.e. three times higher than expected if all age groups would be equally affected by influenza.

All RIRs obtained in the different seasons and countries were pooled into a summary Relative Illness Ratio (sRIR) (separately for each age group) through random-effects meta-analysis models by using the method by DerSimonian and Laird [19]. Comparisons between pairs of sRIRs were conducted, within each age group, through meta-regression models that included an interaction term for the virus (sub)type.

We assessed the between-estimates heterogeneity by using the I^2^ statistics, which can be interpreted as the percentage of total variation that is attributable to heterogeneity [20]. We expected a high degree of heterogeneity (this was an a priori consideration), as many factors combine to determine the age distribution of influenza surveillance cases in any given country and season. In order to assess this point in detail, we fitted meta-regression models and performed sub-group analysis to explore possible correlates of between-estimates heterogeneity, using the proportion of outpatients among reported influenza cases, the percentage of all influenza cases caused by each influenza virus (sub)type in the same or in the previous season, and the country’s latitude, ageing index and per capita GDP.

All analyses were conducted using Stata version 14 (Stata Corp, College Station, TX). We used the *metan* command (with the option *random*) to pool the country- and season-specific RIR into a sRIR, obtain forest plots, and calculate the I^2^ statistics [21]. The study of between-estimates heterogeneity was conducted by using the *metareg* command [21]. Maps were prepared using freely available software (http://mapchart.net/).

All statistical tests were two-sided, and *p*-values of less than 0.05 were considered as statistically significant.

### Comparing the age of influenza cases

The interpretation of sRIRs requires caution. The age distribution of patients infected with any given influenza virus in each season and country is affected by a number of socio-economic factors, including healthcare seeking behaviour, influenza testing by age, and the way primary and hospital care are organized and delivered. The chance of seeking medical attention (and consequently being seen by an influenza surveillance system) once a person is infected is very probably not the same in all age groups across all participating countries. For instance, children and the elderly might be over-represented in both general practitioners- and (especially) hospital-based surveillance systems, as they seek medical attention more frequently when they are sick compared to adults. Therefore, we believe a “same virus, different age groups” approach (i.e. a comparison of sRIRs between people of different age groups infected with the same influenza virus) is not correct. In other words, it is not possible to answer the question “*Are young children more or less affected than adults (or another age group) by influenza and by how much?*” by inferring from the age distribution of cases reported to the influenza surveillance system.

Instead, there is no major obstacle in ranking the sRIRs for people of the same age group who are infected with different influenza virus (sub)types (“different viruses, same age group” approach). As mentioned, there are only minor differences in the clinical presentation of influenza illness produced by the different virus types within each age group [9–12]. This implies that the likelihood of seeking medical attention is likely to be similar for people of any given age group in the same country who are infected with different influenza viruses. It is therefore possible to answer the question “*Are young children more or less affected by influenza B compared to influenza A(H3N2)?”*.

## Results

**Fig. 1:**
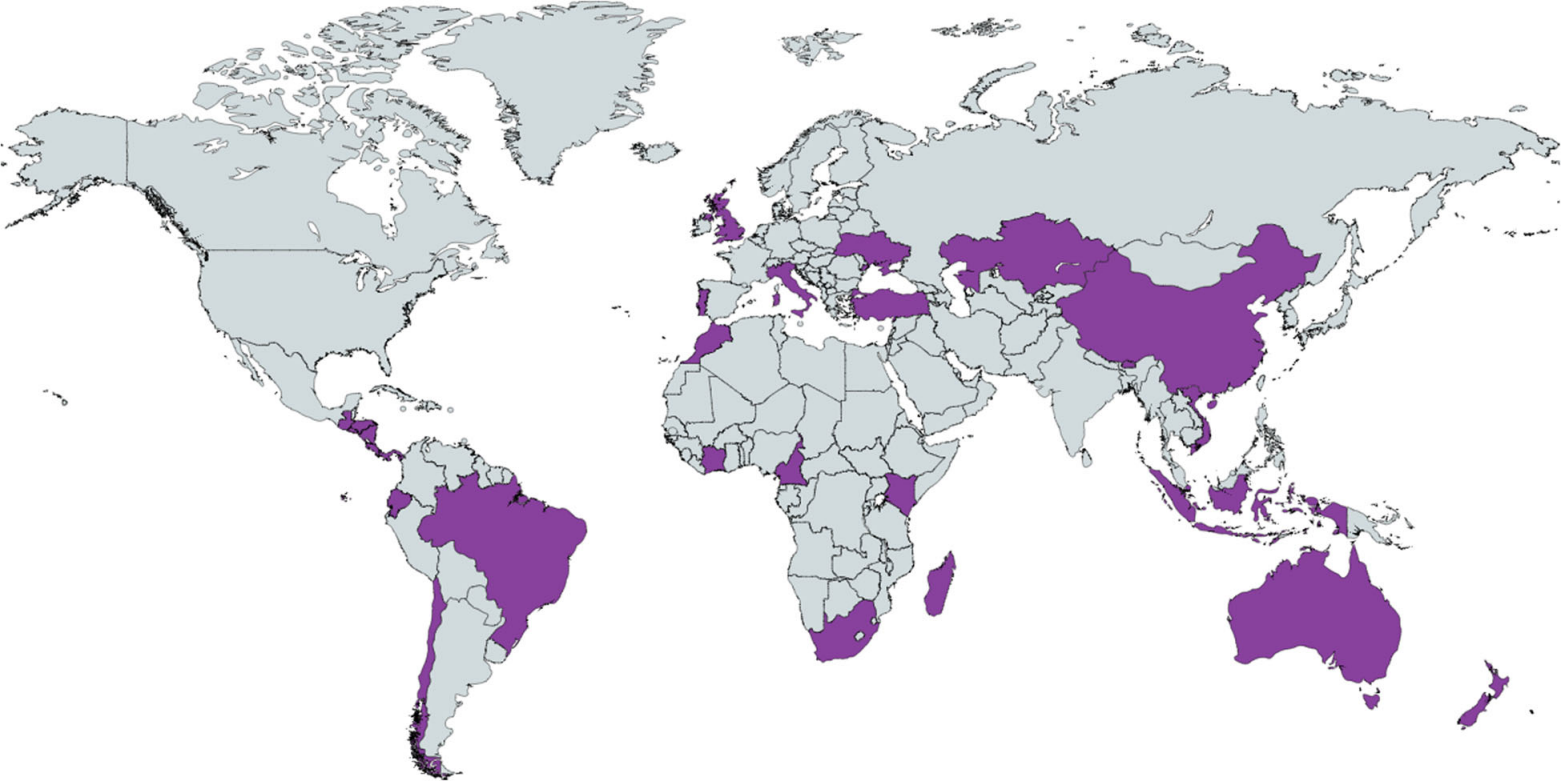
Countries included in the analysis on the age distribution of influenza cases by virus type and subtype. The Global Influenza B Study, 1999-2014. The map was prepared using freely available software (http://mapchart.net/)

The GIBS database that was used for the analysis included 358,768 influenza cases reported between 1999 and 2014 in twenty-nine countries (Table 1 and Fig. 1), of which four were in the Southern hemisphere, fifteen in the inter-tropical belt, and ten in the Northern hemisphere. In detail, there were 19,873 influenza A(H1N1) cases from 33 seasons in twelve countries; 151,685 influenza A(H1N1)pdm2009 cases from 60 seasons in twenty-five countries; 83,791 influenza A(H3N2) cases from 102 seasons in twenty-six countries; and 103,419 influenza B cases from 93 seasons in twenty-six countries. The number of influenza cases (by influenza virus) that was available for the analysis in each country and season (i.e. with age information available and at least 100 cases reported in the season, and after excluding non-subtyped influenza A cases) is reported in the Additional file 1.

**Table 1:** Selected features of countries included in the study of age distribution of influenza cases. The Global Influenza B Study, 1999-2014

Country	Latitude ^(a)^	Population (million)	Ageing index ^(b)^	GDP per capita (USD)	% of outpatients ^(c)^	No. influenza cases (seasons) included in the analysis:			
						A(H1N1)	A(H1N1)pdm2009	A(H3N2)	B
Southern hemisphere									
New Zealand	41.8 S	4.5	0.72	30,400	30%	2,274 (5)	3,763 (3)	4,840 (8)	2,763 (7)
Chile	35.8 S	16.6	0.48	19,100	5%	403 (1)	5,399 (3)	3,540 (3)	897 (2)
South Africa	29.0 S	53.0	0.22	11,500	0%	- (0)	432 (3)	421 (3)	527 (3)
Australia	25.8 S	23.4	0.84	43,000	50%	3,614 (6)	50,096 (4)	13,286 (9)	28,673 (12)
Inter-tropical belt									
Madagascar	19.4 S	21.3	0.08	1,000	100%	109 (1)	1,101 (2)	579 (4)	1,004 (4)
Brazil ^(d)^	10.8 S	201.0	0.32	12,100	NA	- (0)	- (0)	- (0)	665 (4)
Ecuador	2.0 S	15.4	0.24	10,600	0%	- (0)	673 (2)	713 (3)	185 (1)
Indonesia	1.7 S	237.4	0.25	5,200	95%	720 (2)	- (0)	1,397 (4)	1,281 (4)
Kenya	0.4 S	44.4	0.07	1,800	40%	365 (2)	1,285 (3)	1,183 (5)	1,454 (5)
Singapore	1.2 N	5.4	0.63	62,400	100%	- (0)	2,634 (3)	1,436 (3)	1,483 (3)
Cameroon	5.7 N	22.5	0.07	2,400	90%	- (0)	- (0)	259 (2)	102 (1)
Ivory Coast	7.6 N	23.2	0.09	1,800	83%	- (0)	344 (2)	171 (1)	446 (2)
Panama	8.6 N	3.7	0.28	16,500	39%	- (0)	744 (1)	321 (2)	154 (1)
Costa Rica	10.0 N	4.6	0.30	12,900	27%	- (0)	4,069 (2)	1,026 (4)	387 (2)
Nicaragua	12.9 N	6.1	0.16	4,500	43%	- (0)	3,113 (3)	1,089 (4)	699 (3)
El Salvador	13.8 N	6.1	0.25	7,500	22%	- (0)	858 (2)	317 (2)	437 (3)
Honduras	14.8 N	8.2	0.11	4,800	56%	- (0)	656 (2)	277 (2)	- (0)
Guatemala	15.7 N	15.4	0.12	5,300	72%	263 (1)	1,681 (3)	169 (1)	316 (2)
Viet Nam	16.7 N	89.7	0.23	4,000	40%	1,218 (2)	1,368 (3)	2,781 (6)	2,982 (8)
Northern hemisphere									
Bhutan	27.4 N	0.7	0.22	2,600	97%	- (0)	510 (2)	130 (1)	101 (1)
China South ^(e)^	31.1 N	969.4	0.56	9,800	100%	5,530 (5)	40,754 (2)	30,299 (7)	35,910 (8)
Morocco	32.0 N	33.2	0.24	5,500	70%	- (0)	2,076 (2)	354 (2)	- (0)
Turkey	39.0 N	76.7	0.26	15,300	100%	1,298 (2)	- (0)	- (0)	289 (1)
Portugal	39.3 N	10.4	1.17	22,900	97%	- (0)	648 (3)	2,085 (8)	733 (4)
China North ^(e)^	39.5 N	370.9	0.56	9,800	100%	3,900 (5)	22,951 (3)	13,054 (7)	19,956 (7)
Italy	42.9 N	59.9	1.52	29,600	60%	- (0)	3,880 (2)	1,900 (2)	493 (1)
Kazakhstan	48.0 N	17.9	0.28	14,100	5%	- (0)	108 (1)	421 (3)	163 (1)
Ukraine	49.1 N	44.6	1.14	7,400	NA	- (0)	104 (1)	- (0)	- (0)
England ^(f)^	52.3 N	53.0	0.91	37,300	57%	179 (1)	2,438 (3)	1,743 (6)	1,319 (3)
						19,873 (33)	151,685 (60)	83,791 (102)	103,419 (93)

*NA*: not available

^(a)^ Latitude of centroid (when available) or largest city

^(b)^Defined as the number of people aged 65 years or older per hundred people aged 14 years or younger

^(c)^Proportion of outpatients among reported influenza cases

^(d)^Information on subtype of influenza A cases was not available for Brazil

^(e)^Ageing index and GDP per capita of China (whole country)

^(f)^Ageing index and GDP per capita of United Kingdom

The overall age distribution of influenza cases was as follows: 19% in young children, 33% in older children, 30% in young adults, 14% in older adults and 4% in the elderly. The sRIRs for each influenza virus (sub)type by age group are reported in Table 2 (we provide a sample of the corresponding forest plots, namely those for A(H1N1), in the Additional file 2 ). The sRIR for young children was highest for influenza A(H1N1) (3.57, 95%CI 3.00-4.14) and lowest for influenza A(H1N1)pdm2009 (2.93, 95%CI 2.68-3.19); for older children, the sRIR was highest for influenza B (1.69, 95%CI 1.53-1.85) and lowest for influenza A(H3N2) (1.04, 95%CI 0.93-1.14); for both young and older adults, it was highest for influenza A(H1N1)pdm2009 (0.94, 95%CI 0.87-1.01 and 0.62, 95%CI 0.55-0.69, respectively) and lowest for influenza B (0.65, 95%CI 0.59-0.71 and 0.41, 95%CI 0.37-0.45, respectively); for the elderly it was highest for influenza A(H3N2) (0.74, 95%CI 0.66-0.83) and lowest for influenza A(H1N1) (0.16, 95%CI 0.12-0.20). The between-estimates heterogeneity was large, as it exceeded 95% for all of the sRIRs among young children, older children and young adults, and was above 90% for all of the sRIRs calculated among older adults and the elderly (with the only exception of the elderly infected with influenza A(H1N1), where it was 73.9%) (Table 2). Because of the large between-estimates heterogeneity, the summary estimates should not be interpreted in a precise manner, and the reported 95% confidence intervals most likely underestimate the real uncertainty in the data and should be considered with caution.

**Table 2:** Summary Relative Illness Ratio (sRIR), lower and upper 95% confidence intervals (CI), and between-estimates heterogeneity (as measured by the I^2^ statistics) for A(H1N1), A(H1N1)pdm2009, A(H3N2) and B influenza virus within each age group. The Global Influenza B Study, 1999-2014

Influenza virus	sRIR	lower CI ^(a)^	upper CI ^(a)^	I^2^
Young children (0-4 years)				
A(H1N1)	3.57	3.00	4.14	97.5%
A(H1N1)pdm2009	2.28	2.10	2.46	97.8%
A(H3N2)	3.30	2.95	3.64	98.2%
B	2.93	2.68	3.19	97.5%
Older children (5-17 years)				
A(H1N1)	1.36	1.19	1.54	95.2%
A(H1N1)pdm2009	1.23	1.02	1.45	99.5%
A(H3N2)	1.04	0.93	1.14	97.2%
B	1.69	1.53	1.85	98.4%
Young adults (18-39 years)				
A(H1N1)	0.84	0.72	0.96	97.1%
A(H1N1)pdm2009	0.94	0.87	1.01	97.5%
A(H3N2)	0.73	0.68	0.78	96.0%
B	0.65	0.59	0.71	98.1%
Older adults (40-64 years)				
A(H1N1)	0.49	0.41	0.57	95.2%
A(H1N1)pdm2009	0.62	0.55	0.69	98.7%
A(H3N2)	0.59	0.55	0.63	93.5%
B	0.41	0.37	0.45	96.6%
Elderly (65+ years)				
A(H1N1)	0.16	0.12	0.20	73.9%
A(H1N1)pdm2009	0.27	0.24	0.31	94.7%
A(H3N2)	0.74	0.66	0.83	95.2%
B	0.38	0.33	0.43	92.4%

^(a)^Because of the between-estimates heterogeneity being above 50%, the reported 95% CI very likely underestimate the real uncertainty in the data, and should be considered with caution.

Latitude, ageing index, GDP per capita and the proportion of outpatients among reported influenza cases were significantly (*p*<0.05) correlated with sRIRs for most influenza virus (sub)types and age groups (Table 3). Countries’ geographic, demographic, economic and epidemiological characteristics explained a smaller part of between-estimates heterogeneity for A(H1N1) compared to the other influenza virus (sub)types.

**Table 3:** Between-estimates heterogeneity (as measured by the I^2^ statistics) for summary relative illness ratio calculated for each influenza virus within each age group, and variables significantly (*p*<0.05) correlating with them. The Global Influenza B Study, 1999-2014

Influenza virus	I^2^	Variables significantly (p<0.05) correlating with sRIRs					
		Latitude	% of cases accounted for in the same season	% of cases accounted for in the previous season	Ageing index	GDP per capita	% of outpatients ^(a)^
Young children (0-4 years)							
A(H1N1)	97.5%						
A(H1N1)pdm2009	97.8%	✓	✓				✓
A(H3N2)	98.2%				✓	✓	✓
B	97.5%				✓	✓	✓
Older children (5-17 years)							
A(H1N1)	95.2%				✓		
A(H1N1)pdm2009	99.5%	✓	✓				✓
A(H3N2)	97.2%	✓			✓		✓
B	98.4%	✓		✓	✓	✓	✓
Young adults (18-39 years)							
A(H1N1)	97.1%				✓	✓	
A(H1N1)pdm2009	97.5%	✓			✓	✓	
A(H3N2)	96.0%	✓			✓		✓
B	98.1%	✓	✓		✓	✓	
Older adults (40-64 years)							
A(H1N1)	95.2%						
A(H1N1)pdm2009	98.7%			✓			✓
A(H3N2)	93.5%				✓	✓	
B	96.6%	✓	✓		✓	✓	
Elderly (65+ years)							
A(H1N1)	73.9%						
A(H1N1)pdm2009	94.7%	✓			✓	✓	✓
A(H3N2)	95.2%	✓			✓		✓
B	92.4%	✓					✓

GDP: gross domestic product.

^(a)^Proportion of outpatients among reported influenza cases

Inspection of stratified sRIRs (available in the Additional file 3) revealed some additional patterns. Pre-pandemic A(H1N1) was the most frequent virus subtype in the 0-4 years age group only in the Northern hemisphere, while A(H3N2) prevailed in the inter-tropical belt and the Southern hemisphere. The proportion of outpatients among reported cases consistently affected the sRIRs among young children and the elderly (sRIR was highest when the proportion of outpatients accounted for less than 40% in the data), and the country’s demographic structure consistently affected the sRIRs among older children and young adults (sRIR was highest when ageing index >0.50). Importantly, the stratified analysis indicated that the results for countries with 70% or more reported influenza cases from outpatients mirror those for the whole database, with pre-pandemic A(H1N1), B, pandemic A(H1N1)2009 and A(H3N2) being the most frequently detected virus strain among young children, older children, young adults and the elderly, respectively. For the other variables, the pattern of sRIRs across values was inconsistent across influenza virus (sub)types, making it difficult to identify a clear pattern. The between-estimates heterogeneity remained very high (>90%) for most stratified sRIRs, irrespective of the influenza virus (sub)type and age group.

## Discussion

We compared the age distribution of influenza cases across different influenza viruses relative to that of the general population using the GIBS database, which encompasses case-based influenza data from twenty-nine countries during 1999-2014. We found that each influenza virus had a relatively higher frequency in a certain age group: influenza A(H1N1) appeared to be relatively more frequent among young children, B among older children, A(H1N1)pdm2009 among young and older adults, and A(H3N2) among the elderly. These results were confirmed in the analysis limited to countries with most reported influenza cases coming from the outpatient setting. In addition, the RIR was highest for young children and lowest for older adults and the elderly whatever the virus strain examined, suggesting that the young children are the age group most affected by influenza in relation to their size in the country’s population. Importantly, however, because of the possible over-representation of children and elderly people in our study sample and of the large (but expected) heterogeneity, we have restrained ourselves from, and cautioned against, interpreting the point estimates in a precise way. In addition, it is very likely that the confidence intervals underestimate the real uncertainty in the data. As a result, we have limited ourselves to ranking the sRIRs for the different influenza virus (sub)types within any given age group (i.e. following a “different viruses, same age group” approach, as explained), and to drawing more cautious conclusions regarding the results.

Many factors affect the probability of being infected with influenza and of being captured by a national influenza surveillance system in each age group. The factors include epidemiological characteristics like the number of effective contacts among susceptible people, the country’s population density and mobility [22, 23], the typical family composition and the average number of people living in a household [24, 25], and the pattern of contacts between people of different age groups [26–28]. Other important factors include influenza vaccine uptake and how national surveillance systems are structured (e.g. community and/or hospital based). These many factors, and the virus strains that are circulating, interact in a complex way to produce a net effect that defines the cases that are captured by the national surveillance systems. This situation may explain the large degree of heterogeneity in our data, which persisted also when conducting the stratified analysis. Because of this, we opted to (i) analyse our data by age group (to control for some of the factors listed above, e.g. the different probability by age of being tested and therefore being reported to the national surveillance system), (ii) present patterns for each influenza virus (sub)type, and (iii) draw cautious conclusions and avoid giving precise effect estimates.

The main strength of our study is the wealth of data included in the GIBS database and the large diversity of countries in terms of their geographical, socio-economic and epidemiological characteristics. This allowed us to investigate simultaneously the effect of several factors on the age distribution of influenza cases relative to that of the general population, which would not have been possible by using data from a single country (or a few countries that are very similar to one another) or for a limited number of seasons. The large heterogeneity in the data prevented us from quantifying the effect of the virus (sub)type on the age distribution of influenza cases in a precise way, and therefore, to answer in a definitive way our study question. However, the adoption of a meta-analytical approach allowed us to describe the between-estimates heterogeneity both analytically and graphically, and was especially useful in exploring the many factors that contribute to determine the age distribution of influenza cases in any given country and season.

The main limitation of the paper is its reliance on data that was not purposely collected to answer the main study question. Passive influenza surveillance systems only allow inferences regarding medically-attended influenza virus infections, and are inherently unable to capture infected people who do not seek medical care. While this does not represent a limitation for the main goals of surveillance (i.e. early warning of epidemics), the data collected within a passive surveillance system may be sub-optimal for purely research purposes. In particular, influenza surveillance systems differ across countries included in GIBS in terms of the proportion of outpatients compared to all reported influenza cases, which may represent an important source of bias for the present analysis [14]. Reassuringly, our results were confirmed when limiting the analysis to countries in which over 70% of influenza cases were outpatients (i.e. originated from a community-based surveillance system). However, meta-regression and subgroup analysis are not as powerful as an analysis which takes advantage of individual-level data, which is warranted in order to confirm or refute our findings. In general, the “same virus, different age groups” interpretation of results is not possible using passive surveillance data. To allow for a more reliable comparison of influenza burden between age groups using surveillance data, each country would need to adopt a sampling protocol for virological testing that allows the probability to sample a (suspected) influenza case in each age group to be known in advance. Despite being quite expensive, active surveillance based on participatory cohorts would allow a better understanding of influenza epidemiology by providing the opportunity to estimate key epidemiological parameters, including the age distribution of cases and the age-specific attack rate, frequency of complications and mortality [29]. A further limitation of our study is the lack of age data for countries in North America, in particular for United States; in addition, some countries contributed data for only a limited number of seasons. The proportion of influenza patients being typed and subtyped may depend on the patient’s age: this may have introduced a bias whose magnitude, however, should have been attenuated by our decision to analyse the data separately within each age group. Finally, we could not run separate analyses by influenza B virus lineage (Yamagata vs. Victoria), as this information was available in only a small percentage of influenza B cases.

## Conclusions

Global surveillance data for the start of the 21^st^ century suggest that the relative proportion of influenza cases caused by each influenza virus (sub)type may differ by age group. Our results suggest that the mix of circulating influenza viruses is one among several factors that are at play in determining the differing age distribution of influenza surveillance cases, and highlight the importance of presenting disease burden estimates by age group and virus (sub)type.

## Additional files

Additional file 1: **Table S1.** Number of influenza cases caused by the difference influenza viruses that were included in the analysis. The Global Influenza B Study, 1999-2014. (DOCX 24 kb)

Additional file 2: **Figure S1.** Forest plot of the Relative Illness Ratio for patients aged 0-4 years infected with A(H1N1) influenza virus. The Global Influenza B Study, 1999-2014. **Figure S2.** Forest plot of the Relative Illness Ratio for patients aged 5-17 years infected with A(H1N1) influenza virus. The Global Influenza B Study, 1999-2014. **Figure S3.** Forest plot of the Relative Illness Ratio for patients aged 18-39 years infected with A(H1N1) influenza virus. The Global Influenza B Study, 1999-2014. **Figure S4.** Forest plot of the Relative Illness Ratio for patients aged 40-64 years infected with A(H1N1) influenza virus. The Global Influenza B Study, 1999-2014. **Figure S5.** Forest plot of the Relative Illness Ratio for patients aged 65+ years infected with A(H1N1) influenza virus. The Global Influenza B Study, 1999-2014. (DOCX 44 kb)

Additional file 3: **Table S2.** Summary Relative Illness Ratio (sRIR), 95% confidence intervals (95% CI) across age groups and influenza viruses by categories of country ageing index. The Global Influenza B Study, 1999-2014. **Table S3.** Summary Relative Illness Ratio (sRIR), 95% confidence intervals (95% CI) across age groups and influenza viruses by percentage of outpatients among cases reported to the influenza surveillance system. The Global Influenza B Study, 1999-2014. **Table S4**. Summary Relative Illness Ratio (sRIR), 95% confidence intervals (95% CI) across age groups and influenza viruses by country latitude. The Global Influenza B Study, 1999-2014. **Table S5.** Summary Relative Illness Ratio (sRIR), 95% confidence intervals (95% CI) across age groups and influenza viruses by percentage of influenza cases caused by that influenza virus in the same season. The Global Influenza B Study, 1999-2014. **Table S6.** Summary Relative Illness Ratio (sRIR), 95% confidence intervals (95% CI) across age groups and influenza viruses by percentage of influenza cases caused by that influenza virus in the previous season. The Global Influenza B Study, 1999-2014. **Table S7.** Summary Relative Illness Ratio (sRIR), 95% confidence intervals (95% CI) across age groups and influenza viruses by categories of country gross domestic product (GDP) per capita. The Global Influenza B Study, 1999-2014. (DOCX 46 kb)

## Abbreviations

GIBS: Global Influenza B Study
RIR: Relative Illness Ratio
sRIR: Summary Relative Illness Ratio
