# Supplementary material for: Distribution of influenza virus types by age using case-based global surveillance data from twenty-nine countries, 1999-2014
Source: BMC Infect Dis. 2018 Jun 8;18:269. doi: 10.1186/s12879-018-3181-y (PMC5994061; doi:10.1186/s12879-018-3181-y)
Supplement: Supplementary file 1 — Table S1. Number of influenza cases caused by the difference influenza viruses that were included in the analysis. The Global Influenza B Study, 1999-2014. (DOCX 24 kb) [file 12879_2018_3181_MOESM1_ESM.docx]

**Additional file 1: Table S1** Number of influenza cases caused by the difference influenza viruses that were included in the analysis. The Global Influenza B Study, 1999-2014.

| **Country** | **Season** | | **A(H1N1)** | | **A(H1N1)pdm2009** | | | **A(H3N2)** | | **B** |
| --- | --- | --- | --- | --- | --- | --- | --- | --- | --- | --- |
| Australia | 2001 | | 102 | | - | | | - | | 117 |
| Australia | 2002 | | - | | - | | | 276 | | 810 |
| Australia | 2003 | | - | | - | | | 314 | | 102 |
| Australia | 2004 | | - | | - | | | - | | 327 |
| Australia | 2005 | | - | | - | | | - | | 920 |
| Australia | 2006 | | - | | - | | | 117 | | 816 |
| Australia | 2007 | | 442 | | - | | | 884 | | 938 |
| Australia | 2008 | | 188 | | - | | | 365 | | 5029 |
| Australia | 2009 | | 672 | | 37414 | | | 1692 | | 474 |
| Australia | 2010 | | 599 | | 6953 | | | 548 | | 1303 |
| Australia | 2011 | | 1611 | | 5507 | | | 1757 | | 7291 |
| Australia | 2012 | | - | | 222 | | | 7333 | | 10546 |
| Bhutan | 2008-2009 | | - | | - | | | 130 | | 101 |
| Bhutan | 2009-2010 | | - | | 273 | | | - | | - |
| Bhutan | 2010-2011 | | - | | 237 | | | - | | - |
| Brazil | 2008 | | - | | - | | | - | | 126 |
| Brazil | 2009 | | - | | - | | | - | | 131 |
| Brazil | 2010 | | - | | - | | | - | | 213 |
| Brazil | 2011 | | - | | - | | | - | | 195 |
| Cameroon | 2009 | | - | | - | | | 110 | | - |
| Cameroon | 2011 | | - | | - | | | - | | 102 |
| Cameroon | 2012 | | - | | - | | | 149 | | - |
| Chile | 2008 | | 403 | | - | | | - | | - |
| Chile | 2009 | | - | | 3964 | | | - | | - |
| Chile | 2010 | | - | | 618 | | | 2020 | | 296 |
| Chile | 2011 | | - | | 817 | | | 246 | | - |
| Chile | 2012 | | - | | - | | | 1274 | | 601 |
| China North | 2005-2006 | | 1103 | | - | | | 115 | | 234 |
| China North | 2006-2007 | | 590 | | - | | | 977 | | 332 |
| China North | 2007-2008 | | - | | - | | | 597 | | 1206 |
| China North | 2008-2009 | | 1603 | | - | | | 149 | | 391 |
| China North | 2009-2010 | | 495 | | 19629 | | | 4855 | | 7778 |
| China North | 2010-2011 | | 109 | | 2974 | | | 4542 | | 1400 |
| China North | 2011-2012 | | - | | 348 | | | 1819 | | 8615 |
| China South | 2005-2006 | | 607 | | - | | | - | | 815 |
| China South | 2006-2007 | | 960 | | - | | | 1267 | | 537 |
| China South | 2007-2008 | | 107 | | - | | | 1022 | | 1671 |
| China South | 2008-2009 | | 1780 | | - | | | 623 | | 1122 |
| China South | 2009-2010 | | 2076 | | 34769 | | | 9522 | | 13878 |
| China South | 2010-2011 | | - | | 5985 | | | 6148 | | 4784 |
| China South | 2011-2012 | | - | | - | | | 6363 | | 12725 |
| China South | 2012-2013 | | - | | - | | | 5354 | | 378 |
| Costa Rica | 2009 | | - | | 3339 | | | 183 | | - |
| Costa Rica | 2010 | | - | | 730 | | | 471 | | 165 |
| Costa Rica | 2011 | | - | | - | | | 114 | | - |
| Costa Rica | 2012 | | - | | - | | | 258 | | 222 |
| Ecuador | 2011 | | - | | 140 | | | 315 | | - |
| Ecuador | 2012 | | - | | - | | | 168 | | 185 |
| Ecuador | 2013 | | - | | 533 | | | 230 | | - |
| El Salvador | 2009 | | - | | 650 | | | - | | - |
| El Salvador | 2010 | | - | | - | | | 186 | | 121 |
| El Salvador | 2011 | | - | | - | | | - | | 101 |
| El Salvador | 2012 | | - | | 208 | | | - | | 215 |
| El Salvador | 2013 | | - | | - | | | 131 | | - |
| England | 2003-2004 | | - | | - | | | 249 | | - |
| England | 2004-2005 | | - | | - | | | 115 | | - |
| England | 2005-2006 | | - | | - | | | - | | 192 |
| England | 2006-2007 | | - | | - | | | 413 | | - |
| England | | 2007-2008 | | 179 | | - | - | | - | |
| England | | 2008-2009 | | - | | - | 499 | | - | |
| England | | 2009-2010 | | - | | 1335 | - | | - | |
| England | | 2010-2011 | | - | | 1003 | - | | 659 | |
| England | | 2011-2012 | | - | | - | 275 | | - | |
| England | | 2012-2013 | | - | | 100 | 192 | | 468 | |
| Guatemala | | 2009 | | 263 | | 1292 | 169 | | 108 | |
| Guatemala | | 2010 | | - | | 220 | - | | 208 | |
| Guatemala | | 2012 | | - | | 169 | - | | - | |
| Honduras | | 2009 | | - | | 506 | - | | - | |
| Honduras | | 2010 | | - | | 150 | 160 | | - | |
| Honduras | | 2011 | | - | | - | 117 | | - | |
| Indonesia | | 2004 | | - | | - | 217 | | 103 | |
| Indonesia | | 2005 | | - | | - | 295 | | 384 | |
| Indonesia | | 2006 | | 420 | | - | 342 | | 324 | |
| Indonesia | | 2007 | | 300 | | - | 543 | | 470 | |
| Italy | | 2008-2009 | | - | | - | 557 | | - | |
| Italy | | 2009-2010 | | - | | 3206 | - | | - | |
| Italy | | 2010-2011 | | - | | 674 | - | | 493 | |
| Italy | | 2011-2012 | | - | | - | 1343 | | - | |
| Ivory Coast | | 2009 | | - | | - | 171 | | 107 | |
| Ivory Coast | | 2011 | | - | | 239 | - | | 339 | |
| Ivory Coast | | 2012 | | - | | 105 | - | | - | |
| Kazakhstan | | 2011-2012 | | - | | - | 109 | | - | |
| Kazakhstan | | 2012-2013 | | - | | - | 153 | | 163 | |
| Kazakhstan | | 2013-2014 | | - | | 108 | 159 | | - | |
| Kenya | | 2008 | | 101 | | - | 216 | | 220 | |
| Kenya | | 2009 | | 264 | | 513 | 211 | | 316 | |
| Kenya | | 2010 | | - | | 343 | 421 | | 174 | |
| Kenya | | 2011 | | - | | 429 | 108 | | 567 | |
| Kenya | | 2012 | | - | | - | 227 | | 177 | |
| Madagascar | | 2006 | | - | | - | 159 | | - | |
| Madagascar | | 2008 | | 109 | | - | - | | - | |
| Madagascar | | 2009 | | - | | 933 | - | | 261 | |
| Madagascar | | 2010 | | - | | - | 137 | | - | |
| Madagascar | | 2011 | | - | | - | 135 | | 239 | |
| Madagascar | | 2012 | | - | | - | 148 | | 221 | |
| Madagascar | | 2013 | | - | | 168 | - | | 283 | |
| Morocco | | 2004-2005 | | - | | - | 142 | | - | |
| Morocco | | 2009-2010 | | - | | 1950 | - | | - | |
| Morocco | | 2010-2011 | | - | | 126 | - | | - | |
| Morocco | | 2011-2012 | | - | | - | 212 | | - | |
| New Zealand | | 2001 | | 327 | | - | - | | 213 | |
| New Zealand | | 2002 | | - | | - | 340 | | 143 | |
| New Zealand | | 2003 | | - | | - | 806 | | - | |
| New Zealand | | 2004 | | - | | - | 668 | | - | |
| New Zealand | | 2005 | | - | | - | - | | 721 | |
| New Zealand | | 2006 | | - | | - | 382 | | - | |
| New Zealand | | 2007 | | 200 | | - | 281 | | 162 | |
| New Zealand | | 2008 | | - | | - | 325 | | 627 | |
| New Zealand | | 2009 | | 1107 | | 2346 | - | | - | |
| New Zealand | | 2010 | | 538 | | 1274 | - | | - | |
| New Zealand | | 2011 | | - | | - | 465 | | 592 | |
| New Zealand | | 2012 | | 102 | | 143 | 1573 | | 305 | |
| Nicaragua | | 2008 | | - | | - | - | | 152 | |
| Nicaragua | | 2009 | | - | | 2125 | - | | - | |
| Nicaragua | | 2010 | | - | | - | 351 | | 219 | |
| Nicaragua | | 2011 | | - | | 714 | 147 | | - | |
| Nicaragua | | 2012 | | - | | - | 115 | | 328 | |
| Nicaragua | | 2013 | | - | | 274 | 476 | | - | |
| Panama | | 2009 | | - | | 744 | - | | - | |
| Panama | | 2010 | | - | | - | 173 | | - | |
| Panama | | 2012 | | - | | - | - | | 154 | |
| Panama | | 2013 | | - | | - | 148 | | - | |
| Portugal | | 1999-2000 | | - | | - | 279 | | - | |
| Portugal | | 2000-2001 | | - | | - | - | | 109 | |
| Portugal | | 2001-2002 | | - | | - | 234 | | - | |
| Portugal | | 2002-2003 | | - | | - | - | | 210 | |
| Portugal | | 2003-2004 | | - | | - | 497 | | - | |
| Portugal | | 2004-2005 | | - | | - | 319 | | - | |
| Portugal | | 2006-2007 | | - | | - | 271 | | - | |
| Portugal | | 2008-2009 | | - | | - | 222 | | - | |
| Portugal | | 2009-2010 | | - | | 228 | - | | - | |
| Portugal | | 2010-2011 | | - | | 226 | 139 | | 201 | |
| Portugal | | 2011-2012 | | - | | - | 124 | | - | |
| Portugal | | 2012-2013 | | - | | 194 | - | | 213 | |
| Singapore | | 2010 | | - | | 1923 | 763 | | 749 | |
| Singapore | | 2011 | | - | | 498 | 382 | | 273 | |
| Singapore | | 2012 | | - | | 213 | 291 | | 461 | |
| South Africa | | 2009 | | - | | 158 | 192 | | - | |
| South Africa | | 2010 | | - | | - | - | | 199 | |
| South Africa | | 2011 | | - | | 173 | 108 | | 172 | |
| South Africa | | 2012 | | - | | - | 121 | | 156 | |
| South Africa | | 2013 | | - | | 101 | - | | - | |
| Turkey | | 2009-2010 | | 1116 | | - | - | | - | |
| Turkey | | 2010-2011 | | 182 | | - | - | | 289 | |
| Ukraine | | 2012-2013 | | - | | 104 | - | | - | |
| Viet Nam | | 2006 | | 574 | | - | - | | 288 | |
| Viet Nam | | 2007 | | - | | - | 878 | | 275 | |
| Viet Nam | | 2008 | | 644 | | - | 222 | | 621 | |
| Viet Nam | | 2009 | | - | | 696 | 728 | | 460 | |
| Viet Nam | | 2010 | | - | | - | 380 | | 506 | |
| Viet Nam | | 2011 | | - | | 456 | - | | 175 | |
| Viet Nam | | 2012 | | - | | - | 267 | | 398 | |
| Viet Nam | | 2013 | | - | | 216 | 306 | | 259 | |
| **Total** | | **No. season** | | **33** | | **60** | **102** | | **93** | |
|  |  | **No. cases** | | **19873** | | **151685** | **83791** | | **103419** | |

A season was included in the analysis of the age distribution of influenza cases caused by a given influenza virus if there were at least 100 influenza cases caused by that influenza virus in that season.
