# Supplementary material for: Distribution of influenza virus types by age using case-based global surveillance data from twenty-nine countries, 1999-2014
Source: BMC Infect Dis. 2018 Jun 8;18:269. doi: 10.1186/s12879-018-3181-y (PMC5994061; doi:10.1186/s12879-018-3181-y)
Supplement: Supplementary file 2 — Figure S1. Forest plot of the Relative Illness Ratio for patients aged 0-4 years infected with A(H1N1) influenza virus. The Global Influenza B Study, 1999-2014. Figure S2. Forest plot of the Relative Illness Ratio for patients aged 5-17 years infected with A(H1N1) influenza virus. The Global Influenza B Study, 1999-2014. Figure S3. Forest plot of the Relative Illness Ratio for patients aged 18-39 years infected with A(H1N1) influenza virus. The Global Influenza B Study, 1999-2014. Figure S4. Forest plot of the Relative Illness Ratio for patients aged 40-64 years infected with A(H1N1) influenza virus. The Global Influenza B Study, 1999-2014. Figure S5. Forest plot of the Relative Illness Ratio for patients aged 65+ years infected with A(H1N1) influenza virus. The Global Influenza B Study, 1999-2014. (DOCX 44 kb) [file 12879_2018_3181_MOESM2_ESM.docx]

**Figure S1** Forest plot of the Relative Illness Ratio for patients aged 0-4 years infected with A(H1N1) influenza virus. The Global Influenza B Study, 1999-2014.

**Figure S2** Forest plot of the Relative Illness Ratio for patients aged 5-17 years infected with A(H1N1) influenza virus. The Global Influenza B Study, 1999-2014.

**Figure S3** Forest plot of the Relative Illness Ratio for patients aged 18-39 years infected with A(H1N1) influenza virus. The Global Influenza B Study, 1999-2014.

**Figure S4** Forest plot of the Relative Illness Ratio for patients aged 40-64 years infected with A(H1N1) influenza virus. The Global Influenza B Study, 1999-2014.

**Figure S5** Forest plot of the Relative Illness Ratio for patients aged 65+ years infected with A(H1N1) influenza virus. The Global Influenza B Study, 1999-2014.
