# Supplementary material for: Distribution of influenza virus types by age using case-based global surveillance data from twenty-nine countries, 1999-2014
Source: BMC Infect Dis. 2018 Jun 8;18:269. doi: 10.1186/s12879-018-3181-y (PMC5994061; doi:10.1186/s12879-018-3181-y)
Supplement: Supplementary file 3 — Table S2. Summary Relative Illness Ratio (sRIR), 95% confidence intervals (95% CI) across age groups and influenza viruses by categories of country ageing index. The Global Influenza B Study, 1999-2014. Table S3. Summary Relative Illness Ratio (sRIR), 95% confidence intervals (95% CI) across age groups and influenza viruses by percentage of outpatients among cases reported to the influenza surveillance system. The Global Influenza B Study, 1999-2014. Table S4. Summary Relative Illness Ratio (sRIR), 95% confidence intervals (95% CI) across age groups and influenza viruses by country latitude. The Global Influenza B Study, 1999-2014. Table S5. Summary Relative Illness Ratio (sRIR), 95% confidence intervals (95% CI) across age groups and influenza viruses by percentage of influenza cases caused by that influenza virus in the same season. The Global Influenza B Study, 1999-2014. Table S6. Summary Relative Illness Ratio (sRIR), 95% confidence intervals (95% CI) across age groups and influenza viruses by percentage of influenza cases caused by that influenza virus in the previous season. The Global Influenza B Study, 1999-2014. Table S7. Summary Relative Illness Ratio (sRIR), 95% confidence intervals (95% CI) across age groups and influenza viruses by categories of country gross domestic product (GDP) per capita. The Global Influenza B Study, 1999-2014. (DOCX 46 kb) [file 12879_2018_3181_MOESM3_ESM.docx]

**Table S2**. Summary Relative Illness Ratio (sRIR), 95% confidence intervals (95% CI) across age groups and influenza viruses by categories of country ageing index. The Global Influenza B Study, 1999-2014.

| **Ageing index** | **No. Seasons** | **0-4 years** | | | **5-17 years** | | | **18-39 years** | | | **40-64 years** | | | **65+ years** | | |
| --- | --- | --- | --- | --- | --- | --- | --- | --- | --- | --- | --- | --- | --- | --- | --- | --- |
|  |  | **sRIR** | **lower CI ^(a)^** | **upper CI ^(a)^** | **sRIR** | **lower CI ^(a)^** | **upper CI ^(a)^** | **sRIR** | **lower CI ^(a)^** | **upper CI ^(a)^** | **sRIR** | **lower CI ^(a)^** | **upper CI ^(a)^** | **sRIR** | **lower CI ^(a)^** | **upper CI ^(a)^** |
| **A(H1N1)** | | | | | | | | | | | | | | | | |
| **Overall** | 33 | 3.57 | 3 | 4.14 | 1.36 | 1.19 | 1.54 | 0.84 | 0.72 | 0.96 | 0.49 | 0.41 | 0.57 | 0.16 | 0.12 | 0.2 |
| >0.50 | 22 | 3.83 | 3.11 | 4.55 | 1.52 | 1.32 | 1.73 | 0.90 | 0.76 | 1.04 | 0.47 | 0.38 | 0.56 | 0.15 | 0.11 | 0.19 |
| 0.25-0.50 | 5 | 2.62 | 1.43 | 3.81 | 1.28 | 0.91 | 1.65 | 0.87 | 0.46 | 1.29 | 0.55 | 0.26 | 0.83 | 0.2 | 0.01 | 0.4 |
| <0.25 | 6 | 3.45 | 1.97 | 4.92 | 0.87 | 0.57 | 1.16 | 0.56 | 0.33 | 0.8 | 0.53 | 0.31 | 0.75 | 0.29 | 0.15 | 0.43 |
| **A(H1N1)pdm2009** | | | | | | | | | | | | | | | | |
| **Overall** | 60 | 2.28 | 2.1 | 2.46 | 1.23 | 1.02 | 1.45 | 0.94 | 0.87 | 1.01 | 0.62 | 0.55 | 0.69 | 0.27 | 0.24 | 0.31 |
| >0.50 | 24 | 2.1 | 1.84 | 2.35 | 1.56 | 1.24 | 1.89 | 1.14 | 1.07 | 1.21 | 0.65 | 0.55 | 0.75 | 0.18 | 0.14 | 0.21 |
| 0.25-0.50 | 9 | 2.47 | 1.73 | 3.21 | 1.22 | 0.94 | 1.3 | 0.89 | 0.71 | 1.06 | 0.67 | 0.55 | 0.78 | 0.51 | 0.3 | 0.72 |
| <0.25 | 27 | 2.44 | 2.09 | 2.78 | 0.98 | 0.77 | 1.19 | 0.76 | 0.65 | 0.87 | 0.58 | 0.47 | 0.68 | 0.42 | 0.27 | 0.57 |
| **A(H3N2)** | | | | | | | | | | | | | | | | |
| **Overall** | 102 | 3.3 | 2.95 | 3.64 | 1.04 | 0.93 | 1.14 | 0.73 | 0.68 | 0.78 | 0.59 | 0.55 | 0.63 | 0.74 | 0.66 | 0.83 |
| >0.50 | 50 | 3.09 | 2.59 | 3.59 | 1.25 | 1.11 | 1.84 | 0.91 | 0.85 | 0.97 | 0.63 | 0.58 | 0.68 | 0.65 | 0.55 | 0.76 |
| 0.25-0.50 | 18 | 3.08 | 2.28 | 3.88 | 0.93 | 0.8 | 1.05 | 0.71 | 0.57 | 0.84 | 0.65 | 0.53 | 0.76 | 1.16 | 0.7 | 1.62 |
| <0.25 | 34 | 3.71 | 3.19 | 4.23 | 0.77 | 0.63 | 0.91 | 0.46 | 0.39 | 0.53 | 0.51 | 0.42 | 0.6 | 0.77 | 0.58 | 0.97 |
| **B** | | | | | | | | | | | | | | | | |
| **Overall** | 93 | 2.93 | 2.68 | 3.19 | 1.69 | 1.53 | 1.85 | 0.65 | 0.59 | 0.71 | 0.41 | 0.37 | 0.45 | 0.38 | 0.33 | 0.43 |
| >0.50 | 45 | 2.77 | 2.41 | 3.14 | 2.13 | 1.99 | 2.28 | 0.76 | 0.68 | 0.83 | 0.45 | 0.39 | 0.5 | 0.36 | 0.3 | 0.42 |
| 0.25-0.50 | 18 | 2.73 | 2.15 | 3.31 | 1.35 | 1.11 | 1.59 | 0.74 | 0.61 | 0.87 | 0.44 | 0.33 | 0.56 | 0.54 | 0.37 | 0.72 |
| <0.25 | 30 | 3.29 | 2.91 | 3.68 | 1.21 | 0.99 | 1.44 | 0.42 | 0.34 | 0.5 | 0.34 | 0.26 | 0.41 | 0.34 | 0.23 | 0.45 |

^(a)^ Because of the between-estimates heterogeneity being above 50%, the reported 95% CI very likely underestimate the real uncertainty in the data, and should be considered with caution.

**Table S3**. Summary Relative Illness Ratio (sRIR), 95% confidence intervals (95% CI) across age groups and influenza viruses by percentage of outpatients among cases reported to the influenza surveillance system. The Global Influenza B Study, 1999-2014.

| **% of outpatients** | **No. Seasons** | **0-4 years** | | | **5-17 years** | | | **18-39 years** | | | **40-64 years** | | | **65+ years** | | |
| --- | --- | --- | --- | --- | --- | --- | --- | --- | --- | --- | --- | --- | --- | --- | --- | --- |
|  |  | **sRIR** | **lower CI ^(a)^** | **upper CI ^(a)^** | **sRIR** | **lower CI ^(a)^** | **upper CI ^(a)^** | **sRIR** | **lower CI ^(a)^** | **upper CI ^(a)^** | **sRIR** | **lower CI ^(a)^** | **upper CI ^(a)^** | **sRIR** | **lower CI ^(a)^** | **upper CI ^(a)^** |
| **A(H1N1)** | | | | | | | | | | | | | | | | |
| **Overall** | 33 | 3.57 | 3 | 4.14 | 1.36 | 1.19 | 1.54 | 0.84 | 0.72 | 0.96 | 0.49 | 0.41 | 0.57 | 0.16 | 0.12 | 0.2 |
| <40% | 8 | 3.61 | 2.33 | 4.89 | 1.33 | 1.08 | 1.58 | 0.88 | 0.57 | 1.19 | 0.51 | 0.3 | 0.73 | 0.15 | 0.07 | 0.24 |
| 40-70% | 9 | 3.48 | 2.51 | 4.45 | 1.1 | 0.82 | 1.39 | 0.83 | 0.52 | 1.15 | 0.6 | 0.43 | 0.78 | 0.19 | 0.15 | 0.23 |
| >70% | 16 | 3.6 | 2.71 | 4.49 | 1.52 | 1.26 | 1.77 | 0.82 | 0.67 | 0.97 | 0.39 | 0.32 | 0.47 | 0.16 | 0.1 | 0.22 |
| **A(H1N1)pdm2009** | | | | | | | | | | | | | | | | |
| **Overall** | 60 | 2.28 | 2.1 | 2.46 | 1.23 | 1.02 | 1.45 | 0.94 | 0.87 | 1.01 | 0.62 | 0.55 | 0.69 | 0.27 | 0.24 | 0.31 |
| <40% | 20 | 2.9 | 2.43 | 3.37 | 0.94 | 0.71 | 1.16 | 0.89 | 0.75 | 1.03 | 0.67 | 0.57 | 0.78 | 0.46 | 0.31 | 0.6 |
| 40-70% | 19 | 2.19 | 1.89 | 2.5 | 1.26 | 1.04 | 1.49 | 0.92 | 0.79 | 1.05 | 0.62 | 0.55 | 0.7 | 0.26 | 0.19 | 0.32 |
| >70% | 20 | 1.87 | 1.55 | 2.19 | 1.51 | 1.04 | 1.97 | 0.97 | 0.88 | 1.06 | 0.53 | 0.46 | 0.61 | 0.16 | 0.12 | 0.2 |
| **A(H3N2)** | | | | | | | | | | | | | | | | |
| **Overall** | 102 | 3.3 | 2.95 | 3.64 | 1.04 | 0.93 | 1.14 | 0.73 | 0.68 | 0.78 | 0.59 | 0.55 | 0.63 | 0.74 | 0.66 | 0.83 |
| <40% | 34 | 4.01 | 3.47 | 4.55 | 0.79 | 0.67 | 0.92 | 0.62 | 0.54 | 0.7 | 0.56 | 0.5 | 0.63 | 1.22 | 0.94 | 1.49 |
| 40-70% | 30 | 3.07 | 2.62 | 3.51 | 0.99 | 0.85 | 1.14 | 0.74 | 0.61 | 0.86 | 0.62 | 0.54 | 0.7 | 0.77 | 0.59 | 0.96 |
| >70% | 38 | 2.84 | 2.2 | 3.48 | 1.28 | 1.12 | 1.44 | 0.83 | 0.77 | 0.9 | 0.59 | 0.54 | 0.65 | 0.43 | 0.36 | 0.51 |
| **B** | | | | | | | | | | | | | | | | |
| **Overall** | 93 | 2.93 | 2.68 | 3.19 | 1.69 | 1.53 | 1.85 | 0.65 | 0.59 | 0.71 | 0.41 | 0.37 | 0.45 | 0.38 | 0.33 | 0.43 |
| <40% | 28 | 3.55 | 3.15 | 3.95 | 1.43 | 1.12 | 1.74 | 0.61 | 0.51 | 0.7 | 0.41 | 0.33 | 0.48 | 0.49 | 0.37 | 0.61 |
| 40-70% | 24 | 2.58 | 2.28 | 2.89 | 1.67 | 1.4 | 1.94 | 0.61 | 0.45 | 0.76 | 0.47 | 0.39 | 0.56 | 0.54 | 0.43 | 0.64 |
| >70% | 37 | 2.75 | 2.27 | 3.23 | 1.98 | 1.76 | 2.2 | 0.66 | 0.58 | 0.75 | 0.34 | 0.29 | 0.39 | 0.21 | 0.17 | 0.25 |

^(a)^ Because of the between-estimates heterogeneity being above 50%, the reported 95% CI very likely underestimate the real uncertainty in the data, and should be considered with caution.

**Table S4**. Summary Relative Illness Ratio (sRIR), 95% confidence intervals (95% CI) across age groups and influenza viruses by country latitude. The Global Influenza B Study, 1999-2014.

| **Latitude** | **No. Seasons** | **0-4 years** | | | **5-17 years** | | | **18-39 years** | | | **40-64 years** | | | **65+ years** | | |
| --- | --- | --- | --- | --- | --- | --- | --- | --- | --- | --- | --- | --- | --- | --- | --- | --- |
|  |  | **sRIR** | **lower CI ^(a)^** | **upper CI ^(a)^** | **sRIR** | **lower CI ^(a)^** | **upper CI ^(a)^** | **sRIR** | **lower CI ^(a)^** | **upper CI ^(a)^** | **sRIR** | **lower CI ^(a)^** | **upper CI ^(a)^** | **sRIR** | **lower CI ^(a)^** | **upper CI ^(a)^** |
| **A(H1N1)** | | | | | | | | | | | | | | | | |
| **Overall** | 33 | 3.57 | 3 | 4.14 | 1.36 | 1.19 | 1.54 | 0.84 | 0.72 | 0.96 | 0.49 | 0.41 | 0.57 | 0.16 | 0.12 | 0.2 |
| Northern hemisphere | 13 | 4.01 | 2.94 | 5.08 | 1.59 | 1.3 | 1.89 | 0.84 | 0.67 | 1.01 | 0.35 | 0.28 | 0.43 | 0.15 | 0.09 | 0.21 |
| Inter-tropical belt | 8 | 3.03 | 2 | 4.07 | 0.97 | 0.72 | 1.23 | 0.64 | 0.42 | 0.85 | 0.58 | 0.4 | 0.75 | 0.26 | 0.16 | 0.37 |
| Southern hemisphere | 12 | 3.46 | 2.59 | 4.33 | 1.37 | 1.18 | 1.57 | 0.97 | 0.71 | 1.22 | 0.56 | 0.39 | 0.73 | 0.15 | 0.1 | 0.21 |
| **A(H1N1)pdm2009** | | | | | | | | | | | | | | | | |
| **Overall** | 60 | 2.28 | 2.1 | 2.46 | 1.23 | 1.02 | 1.45 | 0.94 | 0.87 | 1.01 | 0.62 | 0.55 | 0.69 | 0.27 | 0.24 | 0.31 |
| Northern hemisphere | 19 | 2.01 | 1.65 | 2.38 | 1.57 | 1.12 | 2.02 | 1.1 | 1.01 | 1.18 | 0.56 | 0.47 | 0.65 | 0.2 | 0.14 | 0.25 |
| Inter-tropical belt | 28 | 2.19 | 1.9 | 2.48 | 1.17 | 0.99 | 1.35 | 0.78 | 0.67 | 0.9 | 0.59 | 0.5 | 0.69 | 0.37 | 0.26 | 0.49 |
| Southern hemisphere | 13 | 2.99 | 2.53 | 3.45 | 0.88 | 0.52 | 1.24 | 1.01 | 0.86 | 1.16 | 0.73 | 0.65 | 0.81 | 0.35 | 0.27 | 0.44 |
| **A(H3N2)** | | | | | | | | | | | | | | | | |
| **Overall** | 102 | 3.3 | 2.95 | 3.64 | 1.04 | 0.93 | 1.14 | 0.73 | 0.68 | 0.78 | 0.59 | 0.55 | 0.63 | 0.74 | 0.66 | 0.83 |
| Northern hemisphere | 36 | 3.15 | 2.49 | 3.81 | 1.43 | 1.27 | 1.59 | 0.87 | 0.8 | 0.94 | 0.56 | 0.5 | 0.61 | 0.45 | 0.39 | 0.52 |
| Inter-tropical belt | 43 | 3.22 | 2.74 | 3.7 | 0.83 | 0.73 | 0.94 | 0.58 | 0.49 | 0.67 | 0.61 | 0.52 | 0.7 | 0.84 | 0.67 | 1.01 |
| Southern hemisphere | 23 | 3.64 | 3.12 | 4.16 | 0.81 | 0.66 | 0.97 | 0.79 | 0.68 | 0.9 | 0.62 | 0.55 | 0.69 | 1.1 | 0.87 | 1.33 |
| **B** | | | | | | | | | | | | | | | | |
| **Overall** | 93 | 2.93 | 2.68 | 3.19 | 1.69 | 1.53 | 1.85 | 0.65 | 0.59 | 0.71 | 0.41 | 0.37 | 0.45 | 0.38 | 0.33 | 0.43 |
| Northern hemisphere | 26 | 3.2 | 2.66 | 3.75 | 2.42 | 2.25 | 2.58 | 0.63 | 0.53 | 0.74 | 0.34 | 0.28 | 0.39 | 0.2 | 0.16 | 0.24 |
| Inter-tropical belt | 43 | 2.97 | 2.56 | 3.38 | 1.37 | 1.19 | 1.55 | 0.56 | 0.47 | 0.66 | 0.34 | 0.28 | 0.4 | 0.31 | 0.24 | 0.39 |
| Southern hemisphere | 24 | 2.55 | 2.29 | 2.82 | 1.46 | 1.12 | 1.8 | 0.81 | 0.74 | 0.88 | 0.59 | 0.52 | 0.67 | 0.63 | 0.54 | 0.72 |

^(a)^ Because of the between-estimates heterogeneity being above 50%, the reported 95% CI very likely underestimate the real uncertainty in the data, and should be considered with caution.

**Table S5**. Summary Relative Illness Ratio (sRIR), 95% confidence intervals (95% CI) across age groups and influenza viruses by percentage of influenza cases caused by that influenza virus in the same season. The Global Influenza B Study, 1999-2014.

| **% of influenza cases caused by that virus in the same season** | **No. Seasons** | **0-4 years** | | | **5-17 years** | | | **18-39 years** | | | **40-64 years** | | | **65+ years** | | |
| --- | --- | --- | --- | --- | --- | --- | --- | --- | --- | --- | --- | --- | --- | --- | --- | --- |
|  |  | **sRIR** | **lower CI ^(a)^** | **upper CI ^(a)^** | **sRIR** | **lower CI ^(a)^** | **upper CI ^(a)^** | **sRIR** | **lower CI ^(a)^** | **upper CI ^(a)^** | **sRIR** | **lower CI ^(a)^** | **upper CI ^(a)^** | **sRIR** | **lower CI ^(a)^** | **upper CI ^(a)^** |
| **A(H1N1)** | | | | | | | | | | | | | | | | |
| **Overall** | 33 | 3.57 | 3 | 4.14 | 1.36 | 1.19 | 1.54 | 0.84 | 0.72 | 0.96 | 0.49 | 0.41 | 0.57 | 0.16 | 0.12 | 0.2 |
| <20% | 12 | 3.37 | 2.62 | 4.12 | 1.29 | 1 | 1.59 | 0.88 | 0.69 | 1.08 | 0.58 | 0.45 | 0.71 | 0.2 | 0.17 | 0.24 |
| 20-80% | 18 | 3.68 | 2.8 | 4.56 | 1.41 | 1.16 | 1.66 | 0.82 | 0.68 | 0.96 | 0.46 | 0.37 | 0.55 | 0.13 | 0.08 | 0.18 |
| >80% | 3 | 3.57 | 0.81 | 6.33 | 1.37 | 0.92 | 1.82 | 0.74 | 0.12 | 1.36 | 0.33 | 0.02 | 0.63 | - | - | - |
| **A(H1N1)pdm2009** | | | | | | | | | | | | | | | | |
| **Overall** | 60 | 2.28 | 2.1 | 2.46 | 1.23 | 1.02 | 1.45 | 0.94 | 0.87 | 1.01 | 0.62 | 0.55 | 0.69 | 0.27 | 0.24 | 0.31 |
| <20% | 4 | 3.36 | 1.63 | 5.09 | 1.04 | 0.2 | 1.88 | 0.97 | 0.55 | 1.39 | 0.76 | 0.27 | 1.26 | 0.31 | 0.07 | 0.55 |
| 20-80% | 44 | 2.41 | 2.16 | 2.66 | 1.14 | 0.84 | 1.43 | 0.93 | 0.85 | 1.02 | 0.61 | 0.53 | 0.68 | 0.2 | 0.16 | 0.24 |
| >80% | 12 | 1.72 | 1.39 | 2.04 | 1.63 | 1.34 | 1.92 | 0.95 | 0.8 | 1.1 | 0.63 | 0.56 | 0.71 | 0.42 | 0.28 | 0.56 |
| **A(H3N2)** | | | | | | | | | | | | | | | | |
| **Overall** | 102 | 3.3 | 2.95 | 3.64 | 1.04 | 0.93 | 1.14 | 0.73 | 0.68 | 0.78 | 0.59 | 0.55 | 0.63 | 0.74 | 0.66 | 0.83 |
| <20% | 17 | 3.51 | 2.94 | 4.08 | 1.22 | 0.92 | 1.52 | 0.71 | 0.59 | 0.84 | 0.52 | 0.44 | 0.6 | 0.57 | 0.42 | 0.71 |
| 20-80% | 68 | 3.37 | 2.92 | 3.82 | 0.96 | 0.85 | 1.06 | 0.7 | 0.64 | 0.76 | 0.59 | 0.54 | 0.64 | 0.81 | 0.68 | 0.95 |
| >80% | 17 | 2.78 | 1.75 | 3.82 | 1.15 | 0.94 | 1.35 | 0.87 | 0.74 | 1 | 0.69 | 0.6 | 0.79 | 0.7 | 0.54 | 0.87 |
| **B** | | | | | | | | | | | | | | | | |
| **Overall** | 93 | 2.93 | 2.68 | 3.19 | 1.69 | 1.53 | 1.85 | 0.65 | 0.59 | 0.71 | 0.41 | 0.37 | 0.45 | 0.38 | 0.33 | 0.43 |
| <20% | 14 | 3.81 | 2.87 | 4.75 | 1.84 | 1.45 | 2.23 | 0.5 | 0.35 | 0.65 | 0.34 | 0.21 | 0.47 | 0.41 | 0.24 | 0.57 |
| 20-80% | 69 | 2.85 | 2.55 | 3.15 | 1.68 | 1.49 | 1.88 | 0.64 | 0.57 | 0.72 | 0.39 | 0.35 | 0.43 | 0.35 | 0.3 | 0.41 |
| >80% | 10 | 2.37 | 1.78 | 2.97 | 1.55 | 1.2 | 1.9 | 0.87 | 0.78 | 0.95 | 0.58 | 0.48 | 0.68 | 0.47 | 0.29 | 0.66 |

^(a)^ Because of the between-estimates heterogeneity being above 50%, the reported 95% CI very likely underestimate the real uncertainty in the data, and should be considered with caution.**Table S6**. Summary Relative Illness Ratio (sRIR), 95% confidence intervals (95% CI) across age groups and influenza viruses by percentage of influenza cases caused by that influenza virus in the previous season. The Global Influenza B Study, 1999-2014.

| **% of influenza cases caused by that virus in the previous season** | **No. Seasons** | **0-4 years** | | | **5-17 years** | | | **18-39 years** | | | **40-64 years** | | | **65+ years** | | |
| --- | --- | --- | --- | --- | --- | --- | --- | --- | --- | --- | --- | --- | --- | --- | --- | --- |
|  |  | **sRIR** | **lower CI ^(a)^** | **upper CI ^(a)^** | **sRIR** | **lower CI ^(a)^** | **upper CI ^(a)^** | **sRIR** | **lower CI ^(a)^** | **upper CI ^(a)^** | **sRIR** | **lower CI ^(a)^** | **upper CI ^(a)^** | **sRIR** | **lower CI ^(a)^** | **upper CI ^(a)^** |
| **A(H1N1)** | | | | | | | | | | | | | | | | |
| **Overall** | 33 | 3.57 | 3 | 4.14 | 1.36 | 1.19 | 1.54 | 0.84 | 0.72 | 0.96 | 0.49 | 0.41 | 0.57 | 0.16 | 0.12 | 0.2 |
| <20% | 16 | 3.57 | 2.74 | 4.4 | 1.27 | 1.01 | 1.53 | 0.83 | 0.66 | 1 | 0.55 | 0.42 | 0.68 | 0.18 | 0.15 | 0.21 |
| 20-80% | 10 | 3.09 | 2.14 | 4.04 | 1.45 | 1.22 | 1.67 | 0.91 | 0.75 | 1.07 | 0.45 | 0.34 | 0.56 | 0.18 | 0.09 | 0.27 |
| >80% | 1 | - | - | - | - | - | - | - | - | - | - | - | - | - | - | - |
| **A(H1N1)pdm2009** | | | | | | | | | | | | | | | | |
| **Overall** | 60 | 2.28 | 2.1 | 2.46 | 1.23 | 1.02 | 1.45 | 0.94 | 0.87 | 1.01 | 0.62 | 0.55 | 0.69 | 0.27 | 0.24 | 0.31 |
| <20% | 29 | 2.19 | 2 | 2.38 | 1.31 | 0.96 | 1.67 | 0.89 | 0.79 | 1 | 0.56 | 0.47 | 0.66 | 0.31 | 0.25 | 0.37 |
| 20-80% | 18 | 2.63 | 2.04 | 3.22 | 1.18 | 1 | 1.36 | 0.91 | 0.77 | 1.05 | 0.58 | 0.49 | 0.67 | 0.21 | 0.17 | 0.25 |
| >80% | 8 | 1.89 | 1.34 | 2.45 | 1.05 | 0.78 | 1.31 | 1.16 | 1.02 | 1.31 | 0.86 | 0.75 | 0.97 | 0.27 | 0.17 | 0.38 |
| **A(H3N2)** | | | | | | | | | | | | | | | | |
| **Overall** | 102 | 3.3 | 2.95 | 3.64 | 1.04 | 0.93 | 1.14 | 0.73 | 0.68 | 0.78 | 0.59 | 0.55 | 0.63 | 0.74 | 0.66 | 0.83 |
| <20% | 42 | 3.39 | 2.93 | 3.85 | 1.09 | 0.92 | 1.25 | 0.71 | 0.63 | 0.78 | 0.59 | 0.53 | 0.64 | 0.76 | 0.63 | 0.9 |
| 20-80% | 45 | 3.54 | 2.96 | 4.13 | 0.97 | 0.82 | 1.12 | 0.71 | 0.64 | 0.78 | 0.57 | 0.51 | 0.64 | 0.72 | 0.58 | 0.86 |
| >80% | 4 | 1.19 | 0.52 | 1.87 | 0.93 | 0.64 | 1.22 | 1.09 | 0.91 | 1.28 | 0.74 | 0.55 | 0.63 | 1.33 | 0.62 | 2.04 |
| **B** | | | | | | | | | | | | | | | | |
| **Overall** | 93 | 2.93 | 2.68 | 3.19 | 1.69 | 1.53 | 1.85 | 0.65 | 0.59 | 0.71 | 0.41 | 0.37 | 0.45 | 0.38 | 0.33 | 0.43 |
| <20% | 33 | 2.83 | 2.31 | 3.34 | 1.89 | 1.59 | 2.19 | 0.66 | 0.55 | 0.77 | 0.38 | 0.32 | 0.44 | 0.36 | 0.28 | 0.44 |
| 20-80% | 43 | 3.14 | 2.81 | 3.46 | 1.61 | 1.39 | 1.83 | 0.59 | 0.5 | 0.67 | 0.42 | 0.37 | 0.48 | 0.36 | 0.29 | 0.43 |
| >80% | 10 | 2.55 | 1.79 | 3.31 | 1.04 | 0.75 | 1.34 | 0.91 | 0.68 | 1.15 | 0.58 | 0.42 | 0.75 | 0.61 | 0.4 | 0.82 |

^(a)^ Because of the between-estimates heterogeneity being above 50%, the reported 95% CI very likely underestimate the real uncertainty in the data, and should be considered with caution.

**Table S7**. Summary Relative Illness Ratio (sRIR), 95% confidence intervals (95% CI) across age groups and influenza viruses by categories of country gross domestic product (GDP) per capita. The Global Influenza B Study, 1999-2014.

| **GDP per capita** | **No. Seasons** | **0-4 years** | | | **5-17 years** | | | **18-39 years** | | | **40-64 years** | | | **65+ years** | | |
| --- | --- | --- | --- | --- | --- | --- | --- | --- | --- | --- | --- | --- | --- | --- | --- | --- |
|  |  | **sRIR** | **lower CI ^(a)^** | **upper CI ^(a)^** | **sRIR** | **lower CI ^(a)^** | **upper CI ^(a)^** | **sRIR** | **lower CI ^(a)^** | **upper CI ^(a)^** | **sRIR** | **lower CI ^(a)^** | **upper CI ^(a)^** | **sRIR** | **lower CI ^(a)^** | **upper CI ^(a)^** |
| **A(H1N1)** | | | | | | | | | | | | | | | | |
| **Overall** | 33 | 3.57 | 3 | 4.14 | 1.36 | 1.19 | 1.54 | 0.84 | 0.72 | 0.96 | 0.49 | 0.41 | 0.57 | 0.16 | 0.12 | 0.2 |
| <7,500 USD | 8 | 3.03 | 3 | 4.14 | 0.97 | 0.72 | 1.23 | 0.64 | 0.42 | 0.85 | 0.58 | 0.4 | 0.75 | 0.26 | 0.16 | 0.37 |
| 7,500-15,000 USD | 10 | 4.9 | 3.96 | 5.83 | 1.77 | 1.45 | 2.09 | 0.7 | 0.52 | 0.88 | 0.28 | 0.22 | 0.35 | 0.13 | 0.07 | 0.19 |
| >15,000 USD | 15 | 2.94 | 2.25 | 3.62 | 1.3 | 1.11 | 1.5 | 1.04 | 0.83 | 1.26 | 0.59 | 0.44 | 0.74 | 0.17 | 0.12 | 0.22 |
| **A(H1N1)pdm2009** | | | | | | | | | | | | | | | | |
| **Overall** | 60 | 2.28 | 2.1 | 2.46 | 1.23 | 1.02 | 1.45 | 0.94 | 0.87 | 1.01 | 0.62 | 0.55 | 0.69 | 0.27 | 0.24 | 0.31 |
| <7,500 USD | 23 | 2.19 | 1.85 | 2.54 | 1.14 | 0.97 | 1.31 | 0.79 | 0.67 | 0.91 | 0.5 | 0.4 | 0.6 | 0.35 | 0.2 | 0.51 |
| 7,500-15,000 USD | 15 | 2.77 | 2.36 | 3.18 | 1.15 | 0.54 | 1.76 | 0.91 | 0.82 | 1 | 0.55 | 0.46 | 0.64 | 0.29 | 0.22 | 0.36 |
| >15,000 USD | 22 | 2.11 | 1.79 | 2.43 | 1.38 | 1.15 | 1.61 | 1.11 | 1 | 1.22 | 0.74 | 0.67 | 0.8 | 0.26 | 0.2 | 0.32 |
| **A(H3N2)** | | | | | | | | | | | | | | | | |
| **Overall** | 102 | 3.3 | 2.95 | 3.64 | 1.04 | 0.93 | 1.14 | 0.73 | 0.68 | 0.78 | 0.59 | 0.55 | 0.63 | 0.74 | 0.66 | 0.83 |
| <7,500 USD | 32 | 3.2 | 2.67 | 3.72 | 0.89 | 0.77 | 1.01 | 0.54 | 0.45 | 0.64 | 0.56 | 0.46 | 0.67 | 0.51 | 0.37 | 0.66 |
| 7,500-15,000 USD | 29 | 4.48 | 3.96 | 5.01 | 1.1 | 0.87 | 1.34 | 0.61 | 0.55 | 0.67 | 0.45 | 0.39 | 0.5 | 0.64 | 0.53 | 0.76 |
| >15,000 USD | 41 | 2.53 | 2.07 | 3 | 1.08 | 0.97 | 1.19 | 0.98 | 0.88 | 1.07 | 0.71 | 0.66 | 0.77 | 0.83 | 0.67 | 0.99 |
| **B** | | | | | | | | | | | | | | | | |
| **Overall** | 93 | 2.93 | 2.68 | 3.19 | 1.69 | 1.53 | 1.85 | 0.65 | 0.59 | 0.71 | 0.41 | 0.37 | 0.45 | 0.38 | 0.33 | 0.43 |
| <7,500 USD | 30 | 2.96 | 2.52 | 3.41 | 1.39 | 1.19 | 1.6 | 0.45 | 0.36 | 0.54 | 0.3 | 0.24 | 0.37 | 0.22 | 0.17 | 0.28 |
| 7,500-15,000 USD | 29 | 4.03 | 3.66 | 4.4 | 1.71 | 1.39 | 2.04 | 0.59 | 0.49 | 0.69 | 0.3 | 0.26 | 0.35 | 0.23 | 0.18 | 0.28 |
| >15,000 USD | 34 | 1.98 | 1.68 | 2.28 | 1.92 | 1.73 | 2.12 | 0.86 | 0.79 | 0.92 | 0.56 | 0.5 | 0.63 | 0.5 | 0.41 | 0.58 |

^(a)^ Because of the between-estimates heterogeneity being above 50%, the reported 95% CI very likely underestimate the real uncertainty in the data, and should be considered with caution.
